# Art Therapy Alleviates the Levels of Depression and Blood Glucose in Diabetic Patients: A Systematic Review and Meta-Analysis

**DOI:** 10.3389/fpsyg.2021.639626

**Published:** 2021-03-12

**Authors:** Qingqi Yang, Qunhui Shao, Qiang Xu, Hui Shi, Lin Li

**Affiliations:** ^1^Department of Dermatology, Air Force Medical Center, Beijing, China; ^2^Department of Cardiovascular Medicine, People's Hospital of Zhongwei, Zhongwei, China; ^3^Department of Health Management Center, People's Hospital of Ningxia Hui Autonomous Region, Yinchuan, China

**Keywords:** art therapy, diabetes, depression, anxiety, blood glucose, meta-analysis

## Abstract

**Objective:** To systematically analyze the effects of art therapy on the levels of depression, anxiety, blood glucose, and glycated hemoglobin in diabetic patients.

**Methods:** We searched Cochrane Library, PubMed, Embase, and ClinicalTrials.gov databases from inception to January 24, 2021. The language of publication was limited to English. Randomized controlled trials (RCTs) that used art therapy to improve mental disorders in diabetic patients were involved. After selection of eligible studies, data were extracted, including the first author's full-name, year of publication, the first author's country of residence, number of intervention and control groups, the mean age of participants, method of intervention, duration of follow-up, and outcome measures. Assessment of quality of the included studies and data extraction were independently carried out by two researchers. RevMan 5.3 software was used to perform statistical analysis.

**Results:** A total of 396 samples from five studies were included, and the eligible studies were RCTs with a parallel design. Methods of art therapy included music therapy and painting therapy. The results showed that compared with the control group, art therapy could positively affect the levels of depression [standardized mean difference (SMD), −1.36; 95% confidence interval (CI), (−1.63, −1.09); *P* < 0.00001] and blood glucose in diabetic patients [mean difference (MD), −0.90; 95% CI, (−1.03, −0.77); *P* < 0.0001], while it had no influence on the levels of anxiety [SMD, −0.31; 95% CI, (−0.93, 0.31); *P* = 0.32] and glycated hemoglobin [MD, 0.22; 95% CI, (−0.02, 0.46); *P* = 0.07].

**Conclusion:** Art therapy may have significant effects on the levels of depression and blood glucose for diabetic patients.

## Introduction

Diabetes mellitus is a group of metabolic disorders characterized by a high blood sugar level over a prolonged period of time. According to the reports released by the International Diabetes Federation (IDF), diabetes has been caused 4.2 million deaths in 2019 worldwide. Besides, more than 1.1 million children and adolescents younger than 20 years old are living with type 1 diabetes (T1D), and the number of adults with diabetes will rise to 700 million in 2045. Simultaneously, the number of cases with type 2 diabetes (T2D) is increasing in several countries, and about 79% of adults suffer from diabetes (Patterson et al., 2019). In addition, diabetes is a chronic disease that requires lifetime medical treatment after diagnosis. In 2019, it was estimated that diabetes-related health expenditure totally reached $760 billion globally for individuals who aged 20–79 years old (Saeedi et al., 2019). Several studies have reported that the incidence of anxiety, depression, and other mental disorders in diabetic patients is significantly higher than that in general population (Hajós et al., 2014; Vancampfort et al., 2016; Smith et al., 2018). Meanwhile, psychological needs of diabetic patients may reduce their compliance with medication, exercise, diet, and other treatment methods, resulting in a poor control of blood glucose level (Anderson et al., 2002). Furthermore, mental disorders, such as depression and anxiety, may increase the risk of complications caused by diabetes. Prospective evidence indicated that the risks of microvascular and macrovascular complications [e.g., foot ulceration, retinopathy, chronic kidney disease (Gonzalez et al., 2010; Iversen et al., 2015; Novak et al., 2016; Khoo et al., 2019), myocardial infarction, and stroke (Lin et al., 2010; Scherrer et al., 2011; Ting et al., 2013; Rådholm et al., 2016)] were elevated in diabetic patients with depression. Therefore, it is highly essential to provide psychotherapy for diabetic patients with mental disorders.

At present, the main psychological treatment for diabetic patients is drug therapy. The 2012 Cochrane Review assessed 19 randomized controlled trials (RCTs) concentrated on pharmacological and psychological interventions for diabetic patients with depression. The results showed that application of psychological and pharmacological interventions can be clinically significant for diabetic patients with depression, and psychiatric medication can moderately control blood glucose level (Baumeister et al., 2014). However, long-term treatments are associated with a number of side effects, such as drowsiness, insomnia, agitation, sexual dysfunction, weight gain, cardiac arrhythmia, and orthostatic hypotension (Pacher and Kecskemeti, 2004). In addition, although psychological consultation has significant clinical efficacy for patients with depression and anxiety, the high cost of consultation makes it unacceptable for the majority of diabetic patients.

Art therapy is a complementary therapy that uses art as a medium to treat behavioral, neurological or mental disorders (Naumburg, 1973). There are several types of art therapy, including visual art therapy, music therapy, dance/movement therapy, drama therapy/psychodrama, etc. Numerous studies (Greco-Vigorito et al., 1996; Wallace et al., 2004; Ozdemir and Akdemir, 2009; Delinsky et al., 2010; Hughes and da Silva, 2011; Afnan and Rosenfeld, 2015; Eum and Yim, 2015; Mandić-Gajić G., 2016; Sarid et al., 2017; Jang et al., 2018; Moghaddasifar et al., 2019) have shown that art therapy is advantageous for patients with post-stroke depression, perinatal mood and anxiety disorders, post-partum depression, post-traumatic stress disorder (PTSD), etc. However, to date, no study has evaluated the effects of art therapy on diabetic patients. The present meta-analysis aimed to assess the effects of art therapy on the psychological status and blood glucose level of diabetic patients, and our findings may provide a reliable reference for the future clinical researches.

## Methods

This systematic review and meta-analysis was conducted in accordance with the PRISMA Statement (Preferred Reporting Items for Systematic Reviews and Meta-analyses), and was registered at PROSPERO (Registration No. CRD42020157752).

### Search Strategy

An online systematic search was performed using Cochrane Library, PubMed, Embase, and ClinicalTrials.gov databases from inception to January 24, 2021. The language of publication was limited to English. The search strategy is shown in Appendix 1. All searches used the combination of medical subject heading terms and free-text terms and were adjusted according to specification of a database.

### Inclusion and Exclusion Criteria

Inclusion criteria were as follows: (1) RCTs; (2) patients who were diagnosed with diabetes (including both T1D and T2D); (3) utilization of a type of art therapy, such as painting, music, dance/movement, or drama; (4) inclusion of indicators of anxiety and depression. Exclusion criteria were as follows: (1) patients with gestational diabetes; (2) incomplete data; (3) duplicate studies.

### Literature Screening, Data Extraction, and Assessment of Risk of Bias

Literature screening, data extraction, and assessment of risk of bias were independently carried out by two researchers. Any discrepancies were resolved through a consensus discussion with a third researcher. After selection of eligible studies, data were extracted, including the first author's full-name, year of publication, the first author's country of residence, number of intervention and control groups, participants' mean age, method of intervention, duration of follow-up, and outcome measures. The Cochrane risk of bias (RoB) assessment tool was herein used to evaluate the overall quality. The selection bias, performance bias, detection bias, attrition bias, reporting bias, and other sources of bias were assessed. The risk of bias for each domain was reported as low, unclear, or high.

### Statistical Analysis

RevMan 5.3 software was used to perform statistical analysis. For analysis of the levels of blood glucose and glycated hemoglobin, mean differences (MDs) with 95% confidence intervals (CIs) were employed. With respect to different psychological assessment methods, for the outcomes of anxiety and depression, standardized mean differences (SMDs) with 95% CIs were used. The *I*^2^ statistic was utilized, describing the percentage of variations across studies due to heterogeneity. Considering the influences of heterogeneity caused by confounding factors on the results, a random-effects model was used to carry out meta-analysis. Subgroup analysis was undertaken to investigate sources of heterogeneity. When at least 10 studies could be involved, the funnel plot and Egger's test were used to assess risk of publication bias.

## Results

### Literature Screening

**Figure 1:**
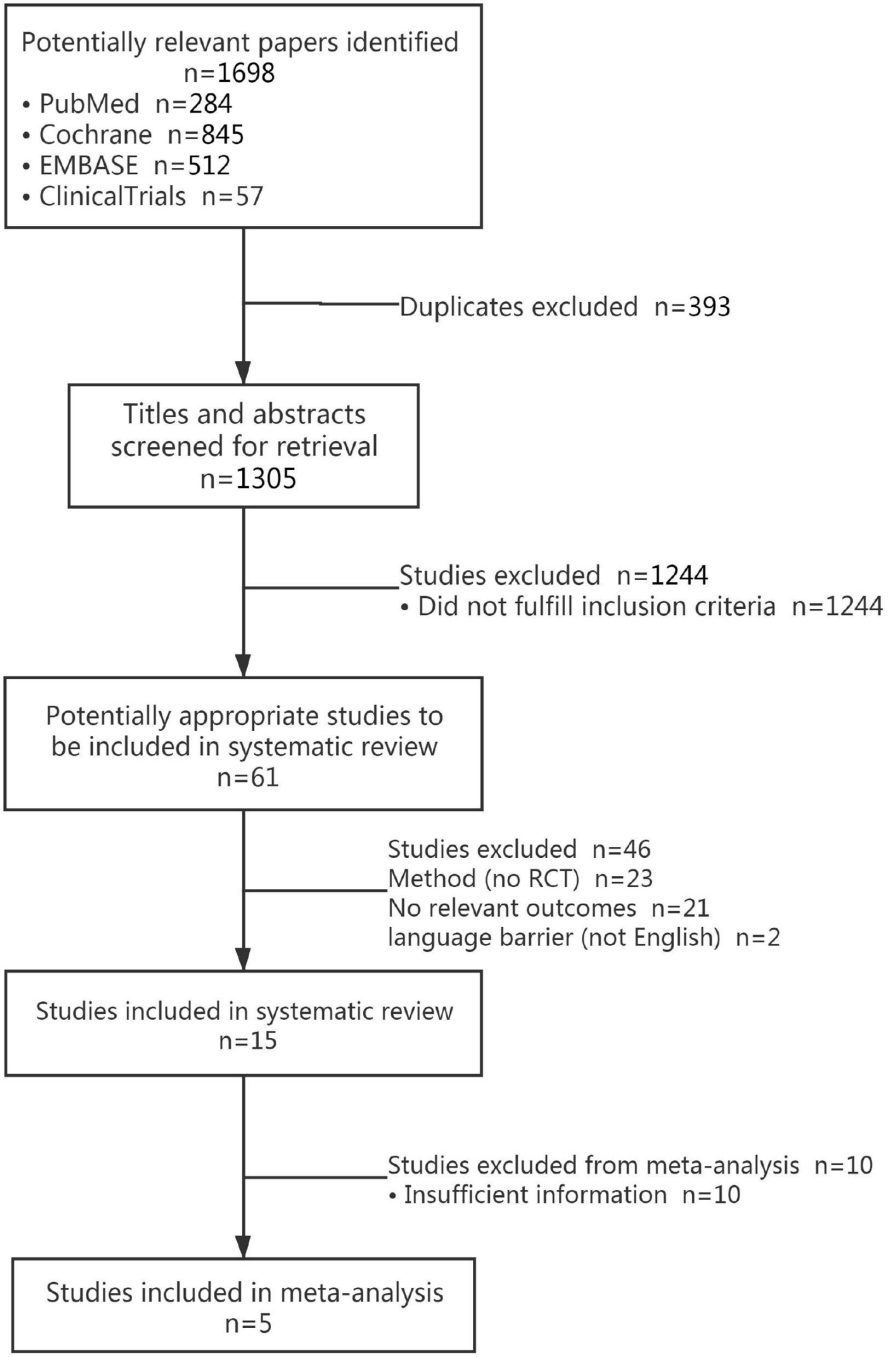
Flowchart of study selection process.

The literature screening resulted in identification of 5 RCTs with inclusion of a total of 396 diabetic patients (Zhao et al., 2005; Harel et al., 2013; Mandel et al., 2013; Eum and Yim, 2015; Singh, 2015; Gelernter et al., 2016; Brandão et al., 2019). Initially, 1,698 studies were screened, of which 393 duplicate studies were excluded using EndNote X9 software. After reading title and abstract of remaining studies, 1,244 articles that did not meet the inclusion criteria were excluded. Besides, 56 studies were removed after reading their full-text, including no RCTs (*n* = 23), no relevant outcomes (*n* = 21), non-English studies (*n* = 2), and incomplete data (*n* = 10). The literature screening process is shown in Figure 1.

### Characteristics of the Included Studies

The characteristics of the included RCTs are summarized in Table 1. It was revealed that five studies were published in China, Israel, the United States, and India from 2005 to 2016. The sample size of the studies ranged from 13 to 182, and the duration of follow-up ranged from 28 days to 2 years. The included studies were RCTs with a parallel design. The methods used in the intervention group were music therapy and painting therapy. Routine nursing was mostly provided to the control group. The outcomes of the studies included blood glucose level, glycated hemoglobin level, State-Trait Anxiety Inventory (STAI), Beck Depression Inventory (BDI), and the Symptom Checklist-90-R (SCL-90-R).

**Table 1:** Characteristics of included studies.

**Study**	**Country**	**Type of diabetes**	**Intervention group**					**Control group**				**Outcome**	**Measurement timepoint (month)**
			**Sample size**	**Age (year)**	**Female (%)**	**Type of Art therapy**	**Intervention frequency**	**Sample size**	**Age (year)**	**Female (%)**	**Intervention**		
Gelernter et al., 2016	Israel	T1DM	7	11.60 ± 3.03	57.14	AGI+BM	Clinic stay 5 days, 1 session/day; 3 months, 1 session/2 weeks, 7 min/session	6	12.17 ± 2.30	66.67	BMS	HbA1c	3
Harel et al., 2013	Israel	T1DM	16	9.3 ± 2.5	75.00	AT	1 session/1–2 weeks for the first 3 months, 1 session/4–6weeks until 9 months, 1 session/8–12 weeks for maintenance therapy	13	9.3 ± 3.4	54.00	SC	BS, HbA1c	24
Mandel et al., 2013	USA	T1DM or T2DM	39	30–85	68.70	MT+DSME/T	1 session/2 weeks, 1.5 h/session	53	30–85	76.60	DSME/T	HbA1c, STAI,	3
Singh, 2015	Indian	T2DM	92	50.4 ± 8.5	44.57	MT	2 sessions/day, 0.5 h/day	90	49.4 ± 8.7	45.46	SC	HbA1c, STAI, BDI	6
Zhao et al., 2005	China	T2DM	40	67.25 ± 5.04	37.50	MT	2 times/day, 0.5 h/times	40	67.43 ± 4.03	37.50	SC	FBS, SCL90	1

*T1DM, type 1 diabetes; T2D, type 2 diabetes; AGI, auditory guided imagery; BM, background music; BMS, background music solely; AT, art therapy; SC, standard care; HbA1c, glycated hemoglobin; BS, blood sugar; FBS, fasting blood sugar; MT, music therapy; DSME/T, diabetes self-management education/training; STAI, state trait anxiety inventory; BDI, beck depression inventory; SCL90, symptom checklist-90*.

### Quality of the Included Studies

**Figure 2:**
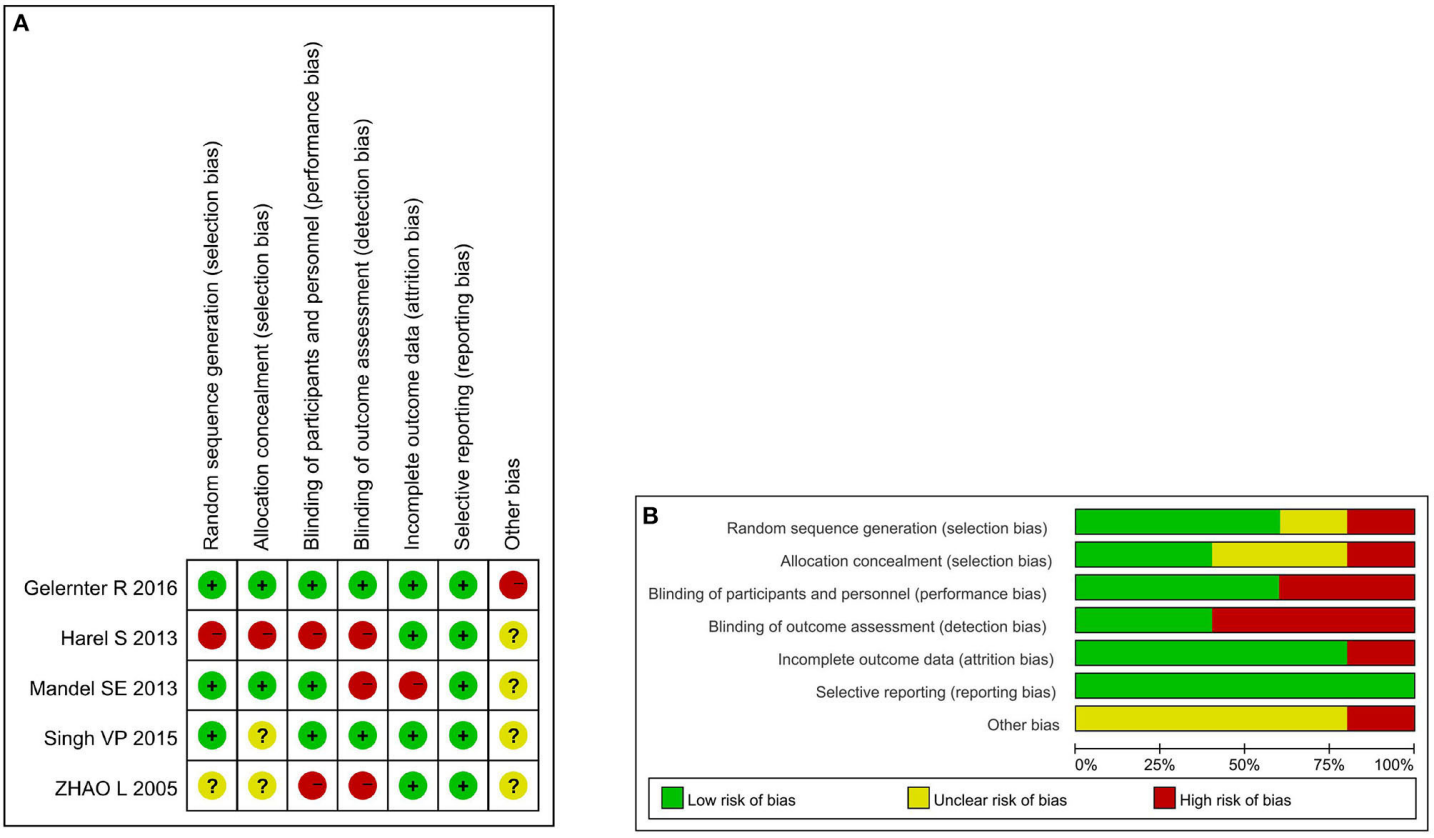
**(A)** Assessment of risk of bias with selected studies. **(B)** Risk of bias graph and summary.

Among five studies, four mentioned “random” in the main text. Besides, three studies adequately generated random allocation sequences (Mandel et al., 2013; Singh, 2015; Gelernter et al., 2016), and one study did not describe the method of generation of random numbers (Zhao et al., 2005). Allocation concealment-associated information was presented in two studies (Mandel et al., 2013; Gelernter et al., 2016). There were two double-blind studies (Singh, 2015; Gelernter et al., 2016), in addition to a single-blind study (Mandel et al., 2013). For incomplete outcome data, only one study had a high-risk of bias as the rate of loss to follow-up was remarkable (33%) (Mandel et al., 2013). All the included studies had a low-risk of reporting bias originated from selective outcome reporting. One study that used guided imagery was suspected of having other sources of bias (Gelernter et al., 2016). The summary of risk of bias is illustrated in Figure 2.

### Meta-Analysis

**Figure 3:**
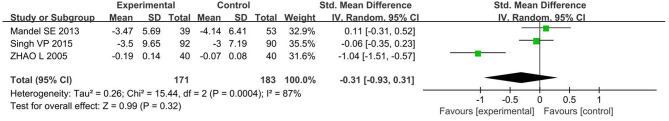
The effects of art therapy on anxiety.

For anxiety-associated outcomes, three studies (Zhao et al., 2005; Mandel et al., 2013; Singh, 2015) that enrolled 354 subjects were included in the meta-analysis. The results of pooled analysis revealed that there was no significant difference in anxiety between intervention and control groups (*P* = 0.32) with a SMD of −0.31 [95% CI, −0.93 to 0.31]. There was a high level of heterogeneity among the included studies (*P* = 0.0004, *I*^2^ = 87%; Figure 3).

**Figure 4:**
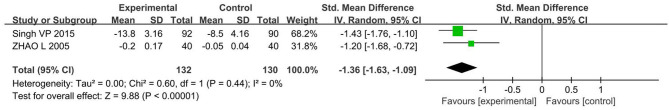
The effects of art therapy on depression.

For depression-associated outcome, two studies (Zhao et al., 2005; Singh, 2015) that enrolled 262 subjects were included in the meta-analysis. The results of pooled analysis showed that there was a significant difference in depression between intervention and control groups (*P* < 0.00001) with a SMD of −1.36 [95% CI, −1.63 to −1.09]. There was no heterogeneity among the included studies (*P* = 0.44, *I*^2^ = 0%; Figure 4).

**Figure 5:**
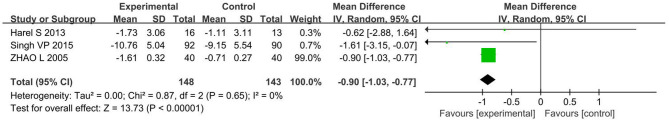
The effects of art therapy on blood glucose level.

For blood glucose level, three studies (Zhao et al., 2005; Harel et al., 2013; Singh, 2015) that enrolled 291 subjects were included in the meta-analysis. The results of pooled analysis indicated a significant difference in blood glucose level between intervention and control groups (*P* < 0.0001) with a MD of −0.90 [95% CI, −1.03 to −0.77]. There was no heterogeneity among the included studies (*P* = 0.65, *I*^2^ = 0%; Figure 5).

**Figure 6:**
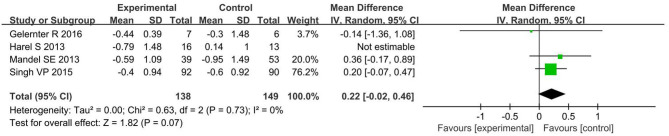
The effects of art therapy on glycated hemoglobin level.

For glycated hemoglobin level, four studies (Harel et al., 2013; Mandel et al., 2013; Singh, 2015; Gelernter et al., 2016) that enrolled 287 subjects were included in meta-analysis. The results of pooled analysis revealed that there was no significant difference in glycated hemoglobin level between intervention and control groups (*P* = 0.07) with a MD of 0.22 [95% CI, −0.02 to 0.46]. There was no heterogeneity among the included studies (*P* = 0.73, *I*^2^ = 0%; Figure 6).

### Subgroup Analysis and Publication Bias

Among the five studies included, four studies used music therapy, only one study employed painting therapy, and subgroup analysis is not meaningful. The publication bias was not assessed, because the number of included RCTs was <10.

## Discussion

This meta-analysis was carried out to comprehensively assess the effects of art therapy on diabetic patients. The analysis of eligible studies showed that art therapy can significantly influence diabetic patients via decreasing blood glucose level and alleviating symptoms of depression, while it has no significant effect on levels of glycated hemoglobin and anxiety. This is the first systematic review on the effects of art therapy on diabetic patients with psychological disorders. Boehm et al. conducted a systematic review and meta-analysis to assess the effects of art therapy on breast cancer patients' levels of anxiety, depression, and quality of life. Their results revealed that art therapy has a positive effect on cancer patients' anxiety, while it has no influence on the level of depression or quality of life (Boehm et al., 2014). Bradt et al. (2015) pointed out that dance/exercise therapy can positively affect cancer patients' levels of depression, stress, anxiety, fatigue, and body image. Tang et al. (2019) demonstrated that art therapy is significant for treating anxiety, depression, and fatigue for patients with breast cancer. Baker et al. (2018) systematically evaluated the effects of art therapy on PTSD. Their findings revealed that symptoms of PTSD were markedly alleviated with the aid of art therapy. Brandão et al. (2019) investigated the effects of art therapy on depression, and their results indicated that art therapy is a safe and reliable therapeutic approach not only for depression, but also for other mental disorders.

In the present systematic review, we found that art therapy can lower blood sugar level, while no previous study has described the underlying mechanism. According to the findings of some related studies, the underlying mechanism may be related to improved function of hypothalamic-pituitary-adrenal (HPA) axis and reduction of cortisol level. During stress, HPA axis is activated. Hypothalamic neurons within the HPA axis secrete corticotropin-releasing hormone that causes the release of adrenocorticotrophic hormone (ACTH) from the pituitary. The ACTH causes the adrenal gland to secrete cortisol (a stress hormone). Together, catecholamines and cortisol increase available sources of energy by promoting lipolysis and the conversion of glycogen into glucose (i.e., blood sugar). D'Cunha et al. (2019) figured out that individuals with dementia are able to improve the function of the HPA axis after undergoing museum therapy. Kaimal et al. (2016) conducted a quasi-experimental study, and investigated the effect of visual art production on the cortisol level of 39 healthy adults, and found that that visual art production could significantly reduce cortisol levels.

Regarding intervention methods, in the current meta-analysis, we noted that the main intervention method was music therapy. However, other types of art therapy possessed certain advantages for the treatment of diabetes, especially visual art therapy. For instance, Isla Pera et al. attempted to indicate whether drawing is significant in the detection of problems associated with psychosocial adaptation in children and adolescents with T1D. Their outcomes showed that the majority of patients had a well-balanced personality, whereas there were also signs of affective or psychosocial difficulties (Isla Pera et al., 2013). Vanelli et al.'s research revealed that painting can promote communication between T1D children and medical staff. Compared with verbal psychotherapy, patients can better perceive diabetes through artistic expression (Vanelli et al., 2018). Moreover, we found that the majority of diabetic patients who had received visual art therapy were adolescents and children with T1D, and there were fewer middle-aged and elderly patients with T2D. This may be related to the fact that art therapy can better facilitate communication with children whose language functions are underdeveloped. However, about 90–95% of diabetic patients suffer from T2D (Deshpande et al., 2008). For such patients, visual art therapy may be a convenient, safe, and easy intervention to manage negative emotions. Therefore, for such patients, especially elderly patients with T2D, it is highly essential to develop further effective classes of art therapy due to the decline in cognitive function, limited activity, vision loss, and increased incidence of complications caused by long-term diseases.

Regardless of newly diagnosed children or elderly people who had been sick for several years, they all should undergo a long-term treatment. In the period of treatment, they are more prone to negative emotions, such as anxiety, stress, depression, loneliness, embarrassment, and isolation. A poor psychological status may affect the control of blood sugar level. Therefore, treatment of diabetes not only requires medical interventions, but also psychological and emotional adjustment. As a complementary therapy, art therapy possesses the advantages of low cost, high safety, relatively fixed teaching methods, easy replication, etc.

The current systematic review contains a number of limitations. Firstly, heterogeneity could not be avoided because of differences in intervention methods, follow-up time, sample size, etc. Secondly, due to the limitation in the number of included documents, we could not perform a meaningful subgroup analysis, which hindered us to understand the effects of art therapy under different conditions. Finally, this systematic review only concentrated on anxiety and depression in diabetic patients, and other clinically meaningful indicators, such as quality of life and social function, were not analyzed, which limited evaluation of the clinical effects of art therapy on diabetic patients.

## Conclusions

The application of art therapy is significant to treat diabetic patients with high levels of blood glucose and depression. However, it is highly essential to include further high-quality RCTs with larger sample size to deeply assess the effects of different types of art therapy on mental health status of diabetic patients.

## Data Availability Statement

The original contributions presented in the study are included in the article/Supplementary Material, further inquiries can be directed to the corresponding author/s.

## Author Contributions

LL designed the study. QY, QS, and LL performed the literature search, article selection, quality appraisal, statistical analysis, and wrote the first draft of the manuscript. QX and HS participated in the revision of the subsequent draft. All authors read and approved the final manuscript.

## Conflict of Interest

The authors declare that the research was conducted in the absence of any commercial or financial relationships that could be construed as a potential conflict of interest.
