# Supplementary material for: Art Therapy Alleviates the Levels of Depression and Blood Glucose in Diabetic Patients: A Systematic Review and Meta-Analysis
Source: Front Psychol. 2021 Mar 12;12:639626. doi: 10.3389/fpsyg.2021.639626 (PMC7994617; doi:10.3389/fpsyg.2021.639626)
Supplement: Supplementary file 1 [file Data_Sheet_1.doc]

Appendix 1. Search strategy

| Literature search (last search performed January 24, 2021) | |
| --- | --- |
| PubMed | ((((((((("randomized controlled trial"[Publication Type] OR "controlled clinical trial"[Publication Type]) OR "randomized"[Title/Abstract]) OR "placebo"[Title/Abstract]) OR "clinical trials as topic"[MeSH Terms:noexp]) OR "randomly"[Title/Abstract]) OR "trial"[Title]) NOT ("animals"[MeSH Terms] NOT ("humans"[MeSH Terms] AND "animals"[MeSH Terms]))) AND ((((("Depression"[MeSH Terms] AND "Depressive Disorder"[MeSH Terms]) OR (("depressi*"[Title/Abstract] OR "dysthymi*"[Title/Abstract]) OR ((("affectiv*"[Title/Abstract] OR "mood"[Title/Abstract]) OR ((("bipolar"[All Fields] OR "bipolarity"[All Fields]) OR "bipolarization"[All Fields]) OR "bipolars"[All Fields])) AND "disorder*"[All Fields]))) OR "Anxiety"[MeSH Terms]) OR "Anxiety Disorders"[MeSH Terms]) OR ((((((((((((((((("agoraphobi*"[Title/Abstract] OR "Anxiety"[Title/Abstract]) OR "anxio*"[Title/Abstract]) OR "phobi*"[Title/Abstract]) OR "panic"[Title/Abstract]) OR "obsessi*"[Title/Abstract]) OR "compulsi*"[Title/Abstract]) OR "OCD"[Title/Abstract]) OR "GAD"[Title/Abstract]) OR "PTSD"[Title/Abstract]) OR "posttrauma*"[Title/Abstract]) OR "post trauma*"[Title/Abstract]) OR "post trauma*"[Title/Abstract]) OR "stress disorder"[Title/Abstract]) OR "neurosis"[Title/Abstract]) OR "neuroses"[Title/Abstract]) OR "neurotic"[Title/Abstract]) OR "psychoneuro*"[Title/Abstract]))) AND (((("Psychodrama"[MeSH Terms] OR ("drama"[Title/Abstract] OR "psychodrama*"[All Fields])) OR (("Dance Therapy"[MeSH Terms] OR (("danc*"[Title/Abstract] OR "movement*"[Title/Abstract]) OR "dance movement"[All Fields])) OR "Primitive expression"[Title/Abstract])) OR ((("Music Therapy"[MeSH Terms] OR "music*"[Title/Abstract]) OR ((("sing*"[Title/Abstract] OR "song*"[Title/Abstract]) OR "choir*"[Title/Abstract]) OR "Rap"[Title/Abstract])) OR ("improvisation*"[Title/Abstract] OR "instrument*"[Title/Abstract]))) OR (("Art Therapy"[MeSH Terms] OR ((("Art"[Title/Abstract] OR "arts"[Title/Abstract]) OR "artist*"[Title/Abstract]) OR "artwork*"[Title/Abstract])) OR (((((((((((("draw*"[Title/Abstract] OR "paint*"[Title/Abstract]) OR "sketch*"[Title/Abstract]) OR "coloring*"[Title/Abstract]) OR "doodle"[Title/Abstract]) OR "doodling"[Title/Abstract]) OR "collage*"[Title/Abstract]) OR "craft*"[Title/Abstract]) OR "sculptur*"[Title/Abstract]) OR "visual*"[Title/Abstract]) OR "expressive*"[Title/Abstract]) OR "Tracing"[Title/Abstract]) OR "still life"[Title/Abstract])))) AND (((("Diabetes Mellitus"[MeSH Terms] OR "Diabetes Complications"[MeSH Terms]) OR "diabet*"[Title/Abstract]) OR (((((("IDDM"[Title/Abstract] OR "NIDDM"[Title/Abstract]) OR "MODY"[Title/Abstract]) OR "T1DM"[Title/Abstract]) OR "T2DM"[Title/Abstract]) OR "T1D"[Title/Abstract]) OR "T2D"[Title/Abstract])) OR (("insulin*"[Title/Abstract] OR "noninsulin*"[Title/Abstract]) AND "depend*"[Title/Abstract])) |
| Cochrane Library | #1 MeSH descriptor: [Diabetes Mellitus] explode all trees  #2 MeSH descriptor: [Diabetes Complications] explode all trees  #3 (diabet*):ti,ab,kw (Word variations have been searched)  #4 ((IDDM OR NIDDM OR MODY OR T1DM OR T2DM OR T1D OR T2D )):ti,ab,kw (Word variations have been searched)  #5 ((insulin* OR noninsulin*) AND (depend*)):ti,ab,kw (Word variations have been searched)  #6 #1 OR #2 OR #3 OR #4 OR #5  #7 MeSH descriptor: [Art Therapy] explode all trees  #8 (Art OR arts OR artist* OR artwork*):ti,ab,kw (Word variations have been searched)  #9 (Draw* OR Paint* OR Sketch* OR coloring* OR doodle OR doodling OR collage* OR craft* OR Sculptur* OR visual* OR expressive* OR Tracing OR "still life"):ti,ab,kw  #10 #7 OR #8 OR #9  #11 MeSH descriptor: [Music Therapy] explode all trees  #12 (Music*):ti,ab,kw  #13 (Sing* OR Song* OR choir* OR Rap):ti,ab,kw  #14 (Improvisation* OR Instrument*):ti,ab,kw  #15 #11 OR #12 OR #13 OR #14  #16 MeSH descriptor: [Dance Therapy] explode all trees  #17 (Danc* OR Movement*):ti,ab,kw  #18 ("Primitive expression"):ti,ab,kw  #19 #16 OR #17 OR #18  #20 MeSH descriptor: [Psychodrama] explode all trees  #21 (drama OR Psychodrama*):ti,ab,kw  #22 #20 OR #21  #23 #10 OR #15 OR #19 OR #22  #24 MeSH descriptor: [Depression] explode all trees  #25 MeSH descriptor: [Depressive Disorder] explode all trees  #26 (Depressi* OR Dysthymi* OR ((affectiv* OR mood OR bipolar) AND disorder*)):ti,ab,kw  #27 MeSH descriptor: [Anxiety Disorders] explode all trees  #28 MeSH descriptor: [Anxiety] explode all trees  #29 (agoraphobi* or anxiety or anxio* or phobi* or panic or obsessi* or compulsi* or OCD or GAD or PTSD or posttrauma* or post-trauma* or post trauma* or stress disorder or neurosis or neuroses or neurotic or psychoneuro*):ti,ab,kw  #30 #24 OR #25 OR #26 OR #27 OR #28 OR #29  #31 #6 AND #23 AND #30  #32 MeSH descriptor: [Clinical Trials as Topic] explode all trees  #33 (randomly):ti,ab,kw OR (randomized controlled trial):pt OR (controlled clinical trial):pt OR (randomized):ti,ab,kw OR (placebo):ti,ab,kw (Word variations have been searched)  #34 #32 OR #33  #35 #31 AND #34 |
| EMBase | ('diabetes mellitus'/exp OR 'diabetic complication'/exp OR diabet*:ab,ti OR (iddm:ab,ti OR niddm:ab,ti OR mody:ab,ti OR t1dm:ab,ti OR t2dm:ab,ti OR t1d:ab,ti OR t2d:ab,ti) OR ((insulin*:ab,ti OR noninsulin*:ab,ti) AND depend*:ab,ti)) AND (('art therapy'/exp OR (art:ab,ti OR arts:ab,ti OR artist*:ab,ti OR artwork*:ab,ti) OR (draw*:ab,ti OR paint*:ab,ti OR sketch*:ab,ti OR coloring*:ab,ti OR doodle:ab,ti OR doodling:ab,ti OR collage*:ab,ti OR craft*:ab,ti OR sculptur*:ab,ti OR visual*:ab,ti OR expressive*:ab,ti OR tracing:ab,ti OR 'still life':ab,ti)) OR ('music therapy'/exp OR music*:ab,ti OR (sing*:ab,ti OR song*:ab,ti OR choir*:ab,ti OR rap:ab,ti) OR (improvisation*:ab,ti OR instrument*:ab,ti)) OR ('dance therapy'/exp OR (danc*:ab,ti OR movement*:ab,ti OR 'dance/movement':ab,ti) OR 'primitive expression':ab,ti) OR ((drama:ab,ti OR psychodrama*:ab,ti) OR 'psychodrama'/exp)) AND ('depression'/exp OR (depressi*:ab,ti OR dysthymi*:ab,ti OR ((affectiv*:ab,ti OR mood:ab,ti OR bipolar:ab,ti) AND disorder*:ab,ti)) OR ('anxiety'/exp OR 'anxiety disorder'/exp) OR (agoraphobi*:ab,ti OR anxiety:ab,ti OR anxio*:ab,ti OR phobi*:ab,ti OR panic:ab,ti OR obsessi*:ab,ti OR compulsi*:ab,ti OR ocd:ab,ti OR gad:ab,ti OR ptsd:ab,ti OR posttrauma*:ab,ti OR 'post trauma*':ab,ti OR 'stress disorder':ab,ti OR neurosis:ab,ti OR neuroses:ab,ti OR neurotic:ab,ti OR psychoneuro*:ab,ti)) AND (('crossover procedure':de OR 'double-blind procedure':de OR 'randomized controlled trial':de) AND or  AND 'single-blind procedure':de OR (random*:de,ab,ti AND or :de,ab,ti AND factorial*:de,ab,ti) OR crossover*:de,ab,ti OR ((cross NEXT/1 over*):de,ab,ti) OR placebo*:de,ab,ti OR ((doubl* NEAR/1 blind*):de,ab,ti) OR ((singl* NEAR/1 blind*):de,ab,ti) OR assign*:de,ab,ti OR allocat*:de,ab,ti OR volunteer*:de,ab,ti) |
